# Investigation of Efficient Alkali Treatment and the Effect of Flame Retardant on the Mechanical and Fire Performance of Frost-Retted Hemp Fiber Reinforced PLA

**DOI:** 10.3390/polym14112280

**Published:** 2022-06-03

**Authors:** Percy Festus Alao, Raimond Press, Heikko Kallakas, Jussi Ruponen, Triinu Poltimäe, Jaan Kers

**Affiliations:** 1 Department of Material and Environmental Technology, Tallinn University of Technology, Ehitajate tee 5, 19086 Tallinn, Estonia; rapres@taltech.ee (R.P.); heikko.kallakas@taltech.ee (H.K.); triinu.poltimae@taltech.ee (T.P.); jaan.kers@taltech.ee (J.K.); 2 Department of Biosystems and Bioproducts, Aalto University, Otakaari 1B, 11000 Aalto, Finland; jussi.ruponen@palonot.com

**Keywords:** hemp fiber, biocomposites, thermocompression, fire behavior, mechanical properties, alkali treatment

## Abstract

This research investigates an effective alkali (NaOH) treatment and fire-retardant coating to produce biocomposites from frost-retted hemp fiber and PLA. The fiber surface treatment with various NaOH concentrations was investigated throughout a range of soaking times. The results show that the extracted non-cellulosic fiber content increases with treatment duration and NaOH concentration, while the fraction of targeted components removed remains nearly unchanged after soaking for 1, 2, and 4 h with a 5 wt.% NaOH solution. At the composite level, the treatment with 5 wt.% NaOH solution for 1 h emerged as the most efficient, with tensile strength, Young’s modulus, flexural strength, and flexural modulus of 89.6 MPa, 9.1 GPa, 121.6 MPa, and 9.6 GPa, respectively, using 30 wt.% fibrous reinforcement. The fire performance of the examined batches of biocomposites improved significantly with the novel fire-retardant (Palonot F1) coating. However, the tensile strength notably decreased, while the flexural properties showed only a slight reduction. In most cases, the biocomposites with the alkali-treated hemp fiber had delayed ignition during the 5 min exposure to the cone heater. The findings in this work contribute to studies that will be required to give design guidelines for sustainable building options.

## 1. Introduction

The necessity to minimize the cost and pollution in the construction, automotive, and other industrial sectors is propelling worldwide biocomposite use and innovation [1], prompting recent legislation and initiatives toward the use of new sustainable materials [2]. As a result, plant-based raw materials such as natural fibers are attracting significant interest due to their low-cost, biodegradability, availability, absorption of CO_2_, and their ability to replace glass fibers or other traditional reinforcement materials in composites [3,4]. In particular, hemp fibers are attracting a lot of attention in Europe for a number of applications [5]. According to Debes et al. [6], the energy required for the production of natural/renewable fiber is 60% less than that of glass fiber. Furthermore, natural fiber reinforced composites (NFRCs) have many advantages, including low density, high specific stiffness, high strength, reduced production cost, a healthy manufacturing process, and minimal tool wear compared to synthetic fiber reinforced composites [7]. However, disadvantages such as low durability, swelling and dimensional instability due to moisture sensitivity, low thermal stability limiting the choice of the polymer matrix, and high flammability are all significant impediments to the use of natural fibers and their composites [8]. In addition to these challenges, there are other concerns such as homogeneity of the natural fibers [9] and poor fire resistance. For instance, the usage of these biocomposites in airplanes, ships, buildings, ground transport, oil and gas facilities, household appliances, and other items needing significant fire resistance should meet strict fire rules [6]. As a result, for biocomposites to be suitable in a variety of applications, their multifunctional properties such as strength, flame retardancy, and resistance to moisture and microorganisms must be considered [10]. 

Chemical or physical treatment of natural fibers has been used in numerous studies to increase performance and minimize many of the issues associated with biocomposite applications [5,11]. The impact of chemical fiber surface treatment on the mechanical and water behavior features of NFRCs has been extensively examined in recent and past research [9,12,13,14,15,16,17,18,19,20,21,22,23,24,25,26]. However, there is limited research output regarding improving fire performance. Although, some studies have provided practical data about the fire resistance characteristics [27,28] or reported findings on the topic [6]. According to Naughton et al. [27], a fire-resistance test is necessary for any construction material to fulfill building standard regulations. Because natural fibers have a low thermal property [29], improving the fire resistance of NFRCs is critical and will provide more potential to use sustainable materials as fire protection in buildings and provide a significant contribution toward carbon neutrality [30]. However, fire-retardant treatment can often result in the degradation of fiber strength, especially following the fiber treatment that causes the removal of lignin-rich middle lamella responsible for the fiber stiffness [5]. 

Hence, this research aims to investigate fiber surface treatment with alkali to design the treatment process that is then combined with fire retardant coating to produce a biocomposite that can also function as a fire barrier. To achieve this, the most effective alkali treatment was examined from various concentrations of NaOH solution and treatment time. The impact of the fiber treatment was then studied at the composite level from 30 and 50 wt.% hemp fiber reinforced polylactide (PLA). PLA was considered in this study because developing a fully renewable composite requires the application of natural fiber reinforcements with a renewable polymer matrix [31]. Regardless, PLA shows promise as a feasible substitute for the petroleum-based polymer matrices often used in composite production [18]. PLA, for example, has nearly twice the mechanical characteristics of polypropylene (PP), with tensile strength and flexural modulus of 51 [17]–60 MPa and 3.4 GPa, respectively, compared to 30 MPa and 1.6 GPa for PP [32]. Furthermore, PLA has high strength and modulus that match or exceed the world’s most prevalent petrochemical-based polymers (polystyrene and poly (ethylene terephthalate)) [33]. However, PLA has poor toughness, low melt viscosity, high cost, and inherent brittleness, limiting its use as a polymer matrix in composite manufacture [8], suggesting that further studies are essential to improve its properties. The biocomposite fire performance was investigated using 50 wt.% reinforcement of novel fire retardant coated untreated hemp and alkali-treated hemp fibers. The high fibrous content was chosen because it allows for a more practical approach, which was also ascertained in a past study [18]. The hemp fibers used in this study were frost-retted, and their biochemical composition and properties have been previously characterized [11]. The research results in the current work are presented as fiber mass loss (%) and Fourier transform infrared (FTIR) to illustrate the fiber structural changes after NaOH pretreatment. The suitable treatment was examined on the composite scale using mechanical (tensile and flexure) tests. Fire retardant was investigated from both the mechanical and fire performance of the biocomposite. This study’s findings provide vital information for the valorization of hemp fibers, underutilized in the Baltic area, as well as the prospect of improving biocomposite fire resistance for building construction applications.

## 2. Materials and Methods

### 2.1. Materials

Table 1 presents the properties of the hemp fiber previously reported [11]. The density of the hemp fiber was determined based on EN ISO 1183-1 with a Mettler Toledo AX and MX/UMX using ethanol (97% concentration) to be (1.26 ± 0.04) gcm^−3^. Staple polylactic acid (PLA) (Ingeo bicomponent bonding fiber SLN2450CM) with a density of 1.24 gcm^−3^ from NatureWorks LLC was used as the polymer matrix. The low melting point, tenacity, length, and tensile elongation at break of the PLA Ingeo fibers are 130–170 °C, 3.6 ± 0.5 gd^−1^, 51 mm, and 6%, respectively. Palonot Oy (Otakaari, Espoo, Finland) provided the fire retardant (F1), which has not been previously introduced for hemp fiber reinforced composites and is sustainable, safe, and non-corrosive. Palonot FR (F1) is a novel protic ionic liquid (ILs)-based FR. It is composed of an aqueous solution of bisphosphonate acid, an alkanol amine, and optionally an alkaline agent. The solvent used was water. F1 contains no substances of health concern and is a sustainable and non-corrosive fire-retardant solution for wood-based panels and natural-fiber-based products. F1 provides the highest B-s1, d0 fire retardancy according to EN 13501-1 while allowing quick and effortless treatment. F1 has a pH range of 4.0 to 7.0. Additionally, it can be applied to protect wood against mold, rot, blue stain, insect attacks and dimensional changes [34].

### 2.2. Methods

#### 2.2.1. Fiber Surface Treatment with Alkali

Before alkali pretreatment, the hemp fibers were carded using a classic drum carder (300 mm batt width and 72 teeth per inch) and dried in the oven at 80 °C for 24 h. Subsequently, the fiber surface treatment was performed on dried hemp fibers by soaking in NaOH solution made from Na granules (CAS: 1310-73-2: “STANCHEM” Sp. Z o.o, Boduszyn, Poland). Table 2 presents the alkali treatments examined. The treatment efficiency at the fiber level was then analyzed using mass loss and FTIR spectroscopy. The 30 min treatment with 1 wt.% NaOH solution was not evaluated in this study because a past study [35] showed that it does not sufficiently extract the fiber’s non-cellulosic constituents.

#### 2.2.2. Fire Retardant (F1) Treatment

**Figure 1:**
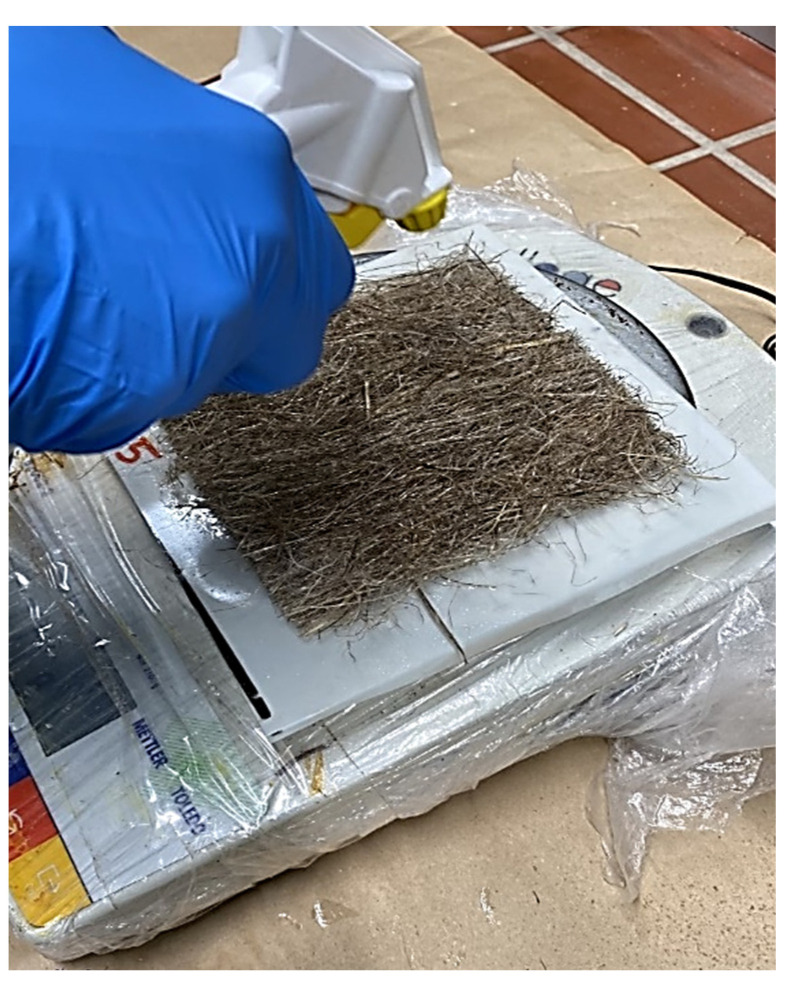
Coating of hemp fiber mats with Palonot fire retardant.

**Figure 2:**
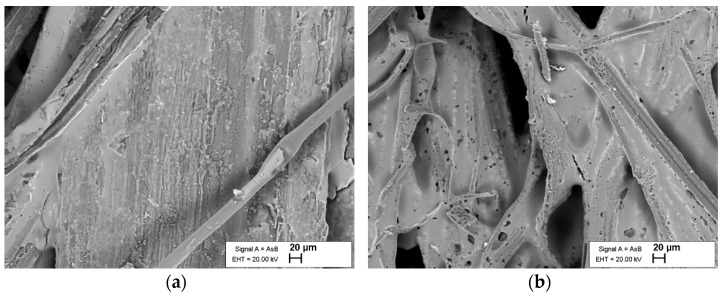
SEM observation of the F1 coating on the hemp fibers: (**a**) untreated, (**b**) alkali-treated.

The F1 was sprayed on the fiber mat, as shown in Figure 1. A total of 40 mL of F1 was applied per board (130 × 100 × 2 mm). The dried matter content of the F1 is roughly 56%. To control the amount of F1 deposited on the fiber surface, the weight of the fiber mat was taken simultaneously as the fire retardant was applied until the mass equivalent of 20 mL F1 was deposited. The coated fiber mats were then oven dried at 40 °C until constant weight. Figure 2 shows the hemp fiber morphology with the F1, observed with a Zeiss Ultra 55 (FELMI-ZFE, Steyrergasse, Austria) scanning electron microscope at 20 kV, depth of 20μm and resolution of 50,000. It is seen that the F1 encapsulated the fiber surface.

#### 2.2.3. Composite Fabrication

Composites of 30 wt.% and 50 wt.% aligned hemp fiber reinforced PLA were fabricated to determine the most effective alkali modification. The biocomposites were manufactured by compression moulding using a 130 × 130 × 2 mm frame with 44 g of hemp and PLA fibers, compressed at 3 MPa and 170–180 °C for 10 min.

**Figure 3:**
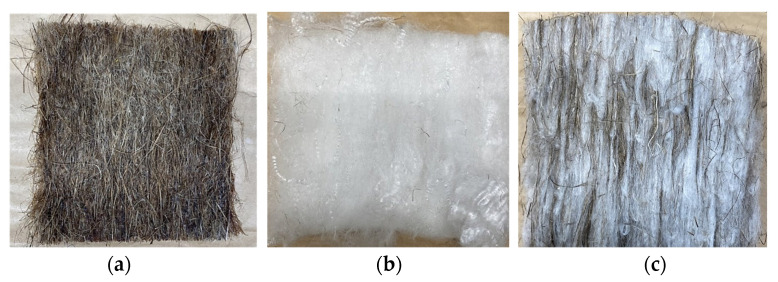
(**a**) Hemp fiber mats with Palonot; (**b**) carded PLA fiber mat; (**c**) carded mix of hemp (50 wt.%) and PLA fibers without F1.

For the F1-coated boards, the biocomposites were about 15–22 g heavier due to the F1 deposited. The F1-coated biocomposites were produced from 11 g top and 11 g bottom mats and 22 g (core of hemp and PLA fibers). The top and bottom mix of the biocomposite were made from 5.5 g of fiber mat sprayed with F1 (Figure 3a) and 5.5 g of PLA mats (Figure 3b). Figure 3c shows the carded 22 g core mix consisting of 11 g of non-F1 treated fibers and 11 g PLA fibers. 

**Figure 4:**
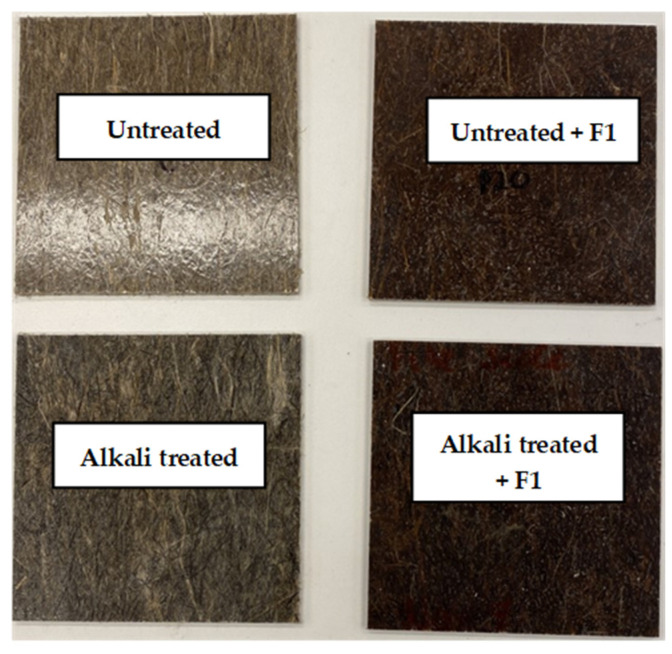
Some batches of the fabricated biocomposites showing the color changes induced by the alkali treatment and F1 coating.

As observed in Figure 4, the alkali treatment changed the color of the resultant composite due to relative structural changes in the hemp fiber following pectin removal. The deposition of F1 resulted in further structural changes in the hemp fibers observed from the loss in fiber flexibility, causing a color change that becomes more apparent after the biocomposite fabrication. Indeed, achieving a consistent color is one of the most difficult criteria for reinforced polymeric composites [36]. The density of all the biocomposites was determined based on EVS-EN ISO 1183-1, with ethanol as the displacement medium.

#### 2.2.4. Fiber Mass Loss

The mass loss (*W*, %) of the hemp fiber following surface treatment with NaOH solution was determined from Equation (1).

(1) $$W\left(\%\right)=\frac{m_{0}-m}{m_{0}}\times 100$$

where *m*_0_ is the weight of the fiber before any treatment, and *m* is the weight of the fiber after alkali treatment.

#### 2.2.5. Fourier Transform Infrared (FTIR)

The FTIR spectroscopy was performed using INTERSPEC 200-X (Spectronic Camspec, Leeds, UK) to assess the fiber surface and fibers after the various alkali treatment. The analysis was carried out directly on the fibers after conditioning in the scanning room for about 7 days. All the FTIR spectra were taken at a resolution of 4 cm^−^^1^. Before scanning the samples, a background scan of a clean Zn–Se diamond crystal was performed. A total of 16 scans on 5 replicas were recorded for each sample.

#### 2.2.6. Mechanical Properties

The tensile and flexural properties of the composites were determined according to EVS-EN ISO 527 (Type 1BA) and EVS-EN ISO 14125:2000 standard tests, respectively, using a Zwick Roell Z050 (August-Nagel-Straße 11, 89079 Ulm, Germany). The test results for each variant are presented as an average of seven (7) replicas with at least 2 test specimens obtained from 3 composite boards. Specimens for the mechanical testing were laser cut at 18 mms^−1^ (for 30 wt.% composites) and 15 mms^−1^ (for 50 wt.% composites).

#### 2.2.7. Fire Protection Properties

The fire behavior of the composite specimen was performed using a cone calorimeter following EN 5660-1:2015. For the test, samples (100 × 100 mm) were subjected to an irradiance based on fully developed fire room conditions (50 kWm^−2^) using a cone heater at 25 mm from the exposed surface. Specimens were conditioned at a temperature of 23 °C and relative humidity of 50% for at least seven (7) days before the test. A birch wood timber block (100 × 100 × 50) mm^3^ was used as the component to be shielded from the fire to determine the potential of the biocomposite to serve as a fire barrier. The mass and thickness of the specimens and birch timber blocks were measured before the test. The timbers were also held in the same condition as the test specimens prior to the experiment. The temperature measured on the surface exposed to the cone heater (t_surf_) and temperature transfer through depth (t_depth_), which is the temperature on the surface in contact with the timber block and the specimen, were measured using two 0.25 mm diameter type K thermocouples (Pentronic AB, Vastervik, Sweden). Three (3) replicas were tested per batch. The experimental outcome is presented as a temperature–time curve at 3 s intervals. The specimens were exposed to the cone heater for 300 s (5 min).

## 3. Results and Discussion

### 3.1. Effect of Fiber Treatment on Weight Reduction

**Figure 5:**
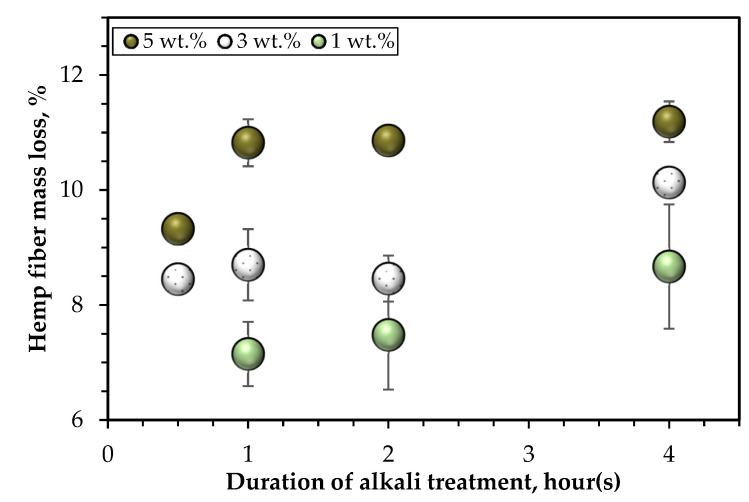
Hemp fiber mass loss following alkali (NaOH) treatment.

Figure 5 presents the hemp fiber mass losses after alkali pretreatments. It is visibly seen that mass loss increased with NaOH concentration and treatment duration. However, there was no meaningful change in the fiber weight when the duration of treatment was increased from 1 to 4 h for the treatment with 5 wt.% NaOH solution. Similarly, fiber treatment with 3 and 1 wt. % NaOH solution resulted in no significant change in fiber weight loss across soaking times of up to 2 h. In addition, there was no significant change in fiber mass reduction after 30 min of treatment with 3 wt.% and 5 wt.% NaOH solutions. The 1-h treatment with a 1 wt.% NaOH solution resulted in the lowest mass loss (7.2 ± 0.6%), while the mass loss increased by about 21% after a 4-h treatment, the result was 17% lower than a 3 wt.% NaOH solution for the same soaking duration. Overall, the most non-cellulosic fiber components (11.2 ± 0.4%) were extracted after immersion for 4 h in 5 wt.% NaOH solution. According to Sair et al. [24], the effective surface area of the hemp fiber increases with the removal of hemicelluloses, lignin, and other amorphous constituents. This implies that to improve the fiber interfacial adhesion with the polymer matrix, the treatment of the hemp fibers with 5 wt.% NaOH solution for 4 h will be the most effective approach. However, since the quantity of non-cellulosic matter extracted with 5 wt.% concentration was almost unchanged at 1, 2 and 4 h of soaking, it appears that it may not be essential to treat the hemp fibers for 4 h and that the 1-h treatment would be the best option in this regard. Furthermore, NaOH treatment tends to weaken the hemp fiber [17] and induce a decrease in stiffness due to lignin removal [37]. Hence, the prolonged soaking of the hemp fibers may cause more degradation of the hemp fiber properties and limit the composites’ mechanical performance. 

### 3.2. FTIR Analysis

**Figure 6:**
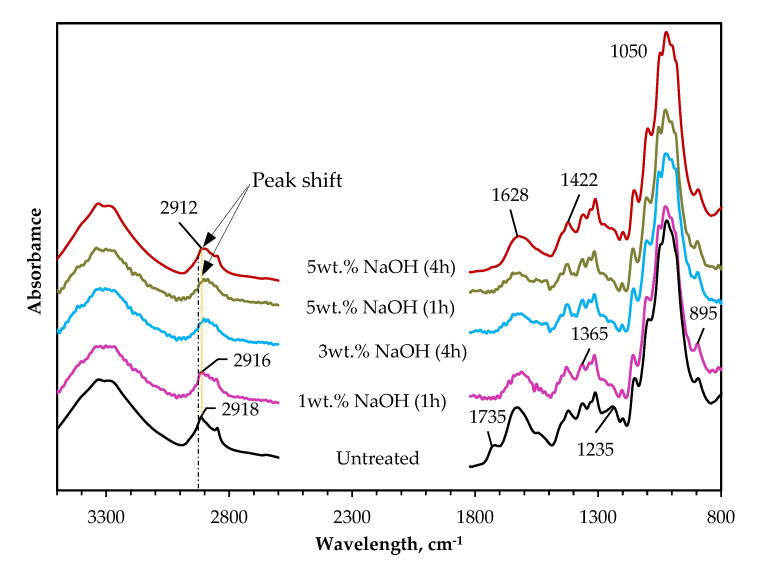
Average vertically shifted spectra of untreated and NaOH pretreated (1 wt.%, 3 wt.%; 5 wt.%) hemp fibers.

Figure 6 shows the average vertically shifted spectra of untreated and alkali-treated hemp fibers (1 wt.%, 3 wt.%, 5 wt.% after 4 h; 5 wt.% after 1 h). These variants of alkali pretreatments are presented in the FTIR because they produced the most fiber weight reduction when considering the specific concentration of NaOH. The spectrum following the 1-h treatment with 5 wt.% NaOH is also shown for comparison with treatment for 4 h. The spectra for the untreated hemp fiber and 4 h alkali treatment with 5 wt.% NaOH solution were previously analyzed and published in a recent article [17]. It is visible from the spectra that the peaks at 895 and 1050 cm^−1^ corresponding to C–H glycosidic bond distortion and C–O stretching vibrations [38] remained unchanged irrespective of the type and duration of the alkali treatment. The peak at 1365 cm^−1^ and that at 1430 cm^−1^ may be due to the C–OH stretching vibrations of the hydrogen bond of crystalline cellulose and C–H bending of amorphous and crystalline cellulose, respectively [39]. These peaks also remained unaffected by the alkali treatment, which implies that the alkali treatments did not affect the cellulose structure of the hemp fibers.

Conversely, with 5 wt.% NaOH solution, there was an attenuation of the peak observed at 2850 cm^−1^ that suggests the removal of a methane group of lignin [40]. A peak reduction and shift were seen at 2916 cm^−1^ after 1-h treatment with 1 wt.% NaOH solution compared to 2918 cm^−1^ shown by the untreated hemp fiber. This shift was more pronounced with higher treatment (3 and 5 wt.%) up to 2912 cm^−1^ for 5 wt.%. Similar notes of peak shifts at this point have been reported in past studies [17,39] and emphasize the removal of the fiber lignin content. The most obvious changes in the hemp fiber structure after the alkali treatments are observed at 1235, 1600–1650, 1516, and 1735 cm^−1^. The peak at 1235 cm^−1^ attributed to the removal of acetyl groups of lignin in the fiber [9,17,39] was reduced after the alkali treatments. The peak at 1516 cm^−1^ corresponding to the vibration of the aromatic skeleton in the lignin [38] was also reduced and in the case of the 4-h 5 wt.% treatment, absent. The peak at 1600–1650 cm^−1^ showing the OH bending mode indicating the presence of water in hemicellulose [39] was attenuated and broadened, while the peak at 1735 cm^−1^ that is caused by the presence of methyl esterified pectins [41] or wax and natural fats [9,17] is also absent after the alkali treatments.

The above observations show that the alkali treatments had some degree of reactivity in removing hemicellulose, lignin, and other non-cellulosic constituents from the hemp fiber. Overall, the treatment with 5 wt.% NaOH solution seems to be the most effective compared to the other alkali treatments and corroborates the fiber mass loss discussed in Section 3.1. 

### 3.3. Effect of Alkali and F1 Treatment on the Mechanical Properties of the Biocomposites

The mechanical performance of the biocomposites was evaluated from fibrous reinforcements of 30 and 50 wt.%. Batches tested include biocomposites reinforced with fibers treated for 1 h with 1 wt.% NaOH solution (AH1), 5 wt.% NaOH solution (AH5) and for 4 h with 5 wt.% NaOH solution (AH5b) compared to untreated fiber reinforced variants (UH). The neat PLA boards were also tested. These specific alkali treatments were selected because the least amount of non-cellulosic fiber contents was extracted from the fiber with 1 wt.% alkali, and the implication of the alkali treatment on the fiber properties in this regard is assumed to be milder compared to the fibers treated for 4 h with 5 wt.% NaOH solution, but the AH5 was tested to investigate the impact of prolonged treatment with 5 wt.% because there were no substantial changes in the amount of targeted components extracted from the fibers for the treatment times of 1, 2 and 4 h.

#### 3.3.1. Tensile Properties

**Figure 7:**
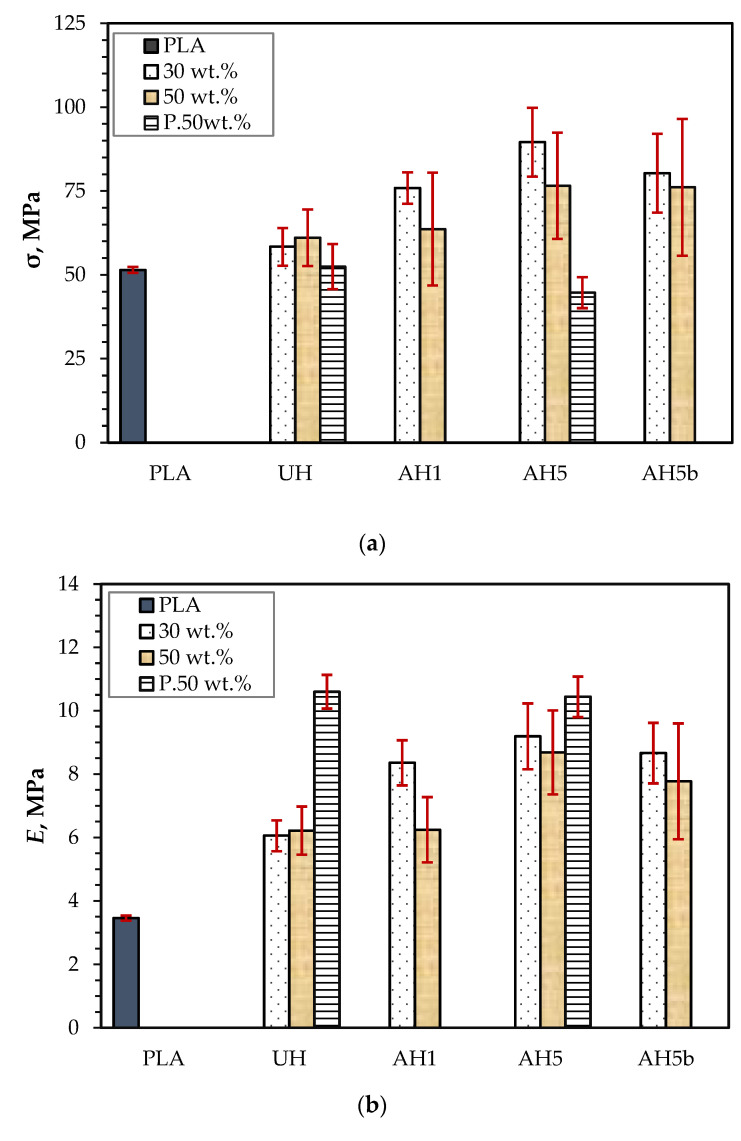
(**a**) Tensile strength (σ) and (**b**) Young’s modulus (*E*) of neat PLA and 30 and 50 wt.% untreated hemp fiber reinforced composites (UH) and composites of alkali-treated hemp fibers (AH1, AH5 and AH5b).

Figure 7a,b show the tensile strength (σ) and Young’s modulus (*E*) of the biocomposites compared to neat PLA [17]. The σ increased slightly following reinforcement of PLA with 30 and 50 wt.% untreated hemp fibers. However, UH showed no meaningful variation in σ at 30 and 50 wt.%. As anticipated, alkali treatments increased the σ of the biocomposites as well, although with 50 wt.% there was no considerable improvement. The σ of the biocomposites was generally much higher using fibrous reinforcement of 30 wt.% than 50 wt.% because there was sufficient wetting of the hemp fibers by the PLA matrix [14,17,18,42,43,44], which ensured better interfacial adhesion [17,43,45]. For the same reason, there was a high standard error and no substantial improvement in the σ of biocomposites with 50 wt.% alkali-treated hemp fibers because the alkali treatment causes a reduction in the fiber diameter due to the removal of middle lamella content, which causes the number of hemp fibers per volume to increase compared to the untreated biocomposites, resulting in more pronounced insufficient fiber wetting [46]. At 30 wt.%, AH5 showed the best σ (90 ± 10 MPa), such that it was 11% higher than that of AH5b, though not significantly. When comparing the average value of composites reinforced with alkali-treated hemp fibers, the σ was generally lower for composites of AH1. The low σ for AH1 compared to AH5 and AH5b may also be due to poor interfacial adhesion between the fiber and PLA matrix resulting from more available non-cellulosic content. The higher σ for AH5 compared to AH5b could be ascribed to the slightly higher (3.4%) removal of non-cellulosic content from the hemp fibers in AH5b. Suardana et al. [47] also showed that the mechanical properties of hemp fiber reinforced composites decreased following the fiber treatment with 6% alkali compared to 4 and 2%, which was attributed to the extraction of more hemicellulose and lignin. Regarding the performance at 50 wt.%, AH5 also presented the highest σ (77 ± 16 MPa), which was only 0.6% more than AH5b. The lowest σ was (61 ± 8 MPa) by UH, which was still roughly 20% more than neat PLA and 4% less than AH1. 

The biocomposites produced *E*, with a similar trend as the σ (Figure 7b). AH5 had the highest *E* (9.2 GPa) with 30 wt.% reinforcement, 5.8% more than AH5b with similar fiber content. AH5 also showed a 10% improvement in *E* at 50 wt.% compared to AH5b. This outcome may be due to the higher reduction in the hemp fiber’s stiffness due to the removal of higher lignin content [17] with 5% alkali treatment for 4 h. UH had the lowest *E* (6.1 GPa) at 30 wt.%, which was still 75% higher than neat PLA. There was no marked difference between the outcomes at 30 wt.% for AH1, AH5 and AH5b and 50 wt.% for UH and AH1. The general improvement in performance for alkali biocomposite variants is due to the substantial removal of the hemicelluloses and lignin from the hemp fiber, which increased the fiber surface roughness and enhanced the bonding between the hemp fibers and the PLA matrix [46,48]. Overall, the tensile performance for both fiber contents show that treatment of the hemp fibers for 1 h with a 5 wt.% NaOH solution appears to be the most efficient. 

**Figure 8:**
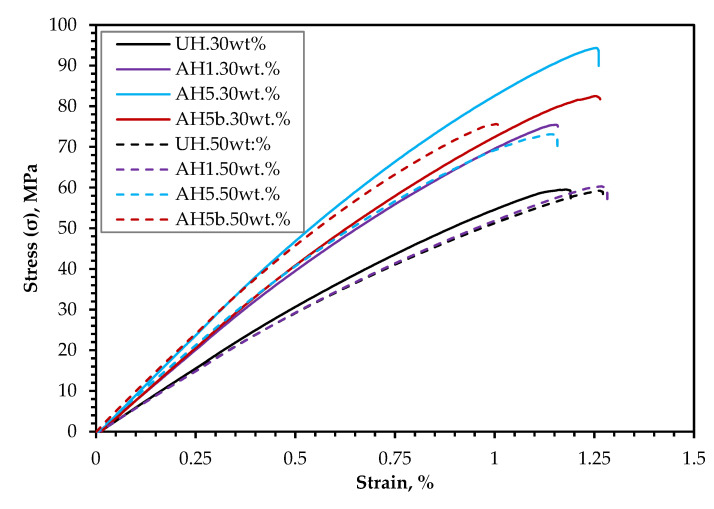
Stress–strain curves of the biocomposites at 30 (–) and 50 wt.% (- -) fiber content.

Figure 8 depicts a typical tensile stress–strain curve of the hemp-PLA composites. There was no discernible yield or hardening phase in the curves. The reinforcement of the PLA with hemp fibers reduced the strain at break for all specimens because the tensile elongation at break of the neat PLA board under tension was found to be (3.03 ± 0.9)%. Hu and Lim [49] similarly observed a lack of yielding point and decrease in strain at break, ascribed to the biocomposites’ seemingly brittle nature under applied force. 

#### 3.3.2. Flexural Properties

**Figure 9:**
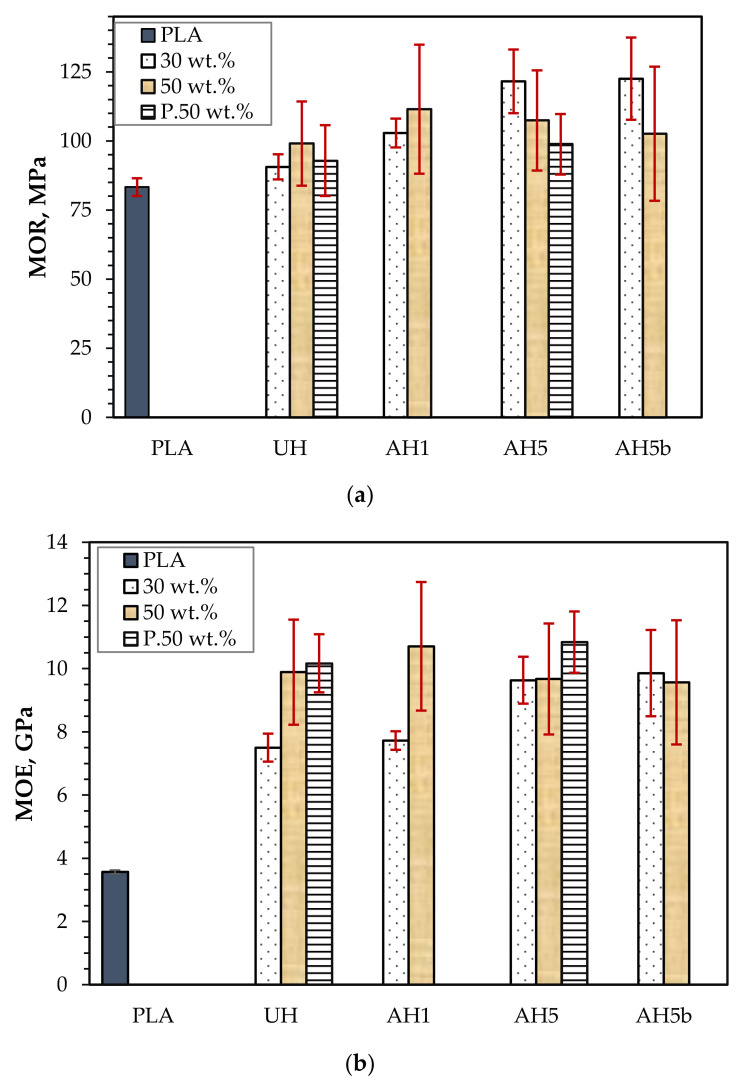
(**a**) MOR and (**b**) MOE of neat PLA and 30 and 50 wt.% untreated hemp fiber reinforced composites (UH) and composites of alkali-treated hemp fibers (AH1, AH5 and AH5b).

The flexural performance of the biocomposites also presented a similar trend to the tensile performance. Figure 9a,b show that the flexural strength (MOR) and modulus (MOE) of AH5 and AH5b are similar, but the 30 wt.% AH5b composites had the highest MOR (123 MPa), which was roughly 1% more than AH5, and 35% higher than UH. Compared to the results of the neat PLA, the reinforcement of the composite with untreated hemp fibers somewhat improved MOR by 8.7% at 30 wt.% and 15.6% at 50 wt.%. UH shows an improved outcome at 50 wt.%, but no significant difference was discernable from statistically comparing the result with 30 wt.%. Likewise, AH1 presented a higher outcome at 50 wt.% compared to AH5 and AH5b, which, as discussed in Section 3.3.1, could be due to the possible insufficient wetting of the hemp fibers. Indeed, the density of hemp fibers reportedly decreased following alkali treatment, which was relative to the concentration of alkali used during treatment [47]. Thus, because of more agglomerated fibers with larger diameters [11] and less fiber–fiber contact, UH experienced lower stress concentration and better fiber wetting than AH1, AH5, and AH5b at 50 wt.%. However, the flexural performance of AH1 was higher than UH, AH5, and AH5b at 50 wt.%, possibly due to fewer bundled fibers in AH1 than in UH, which results in a larger effective surface area for polymer matrix bonding that enhances interfacial adhesion. Moreover, AH5 and AH5b nevertheless produced slightly better outcomes at 50 wt.% than UH, suggesting improved bonding between the hemp fiber and the PLA matrix. Conversely, compared to AH5 and AH5b, AH1 still had less fiber-to-fiber contact. Regarding MOE (Figure 9b), all the composites produced an unchanged outcome at 50 wt.%, but AH5 and AH5b had a significantly higher performance at 30 wt.% than UH and AH1. The MOE of UH and AH1 is representative of the results obtained in MOR, performing better at 50 wt.%, but there was no difference in the MOE for the composite batches of AH5 and AH5b at 30 and 50 wt.%. 

**Figure 10:**
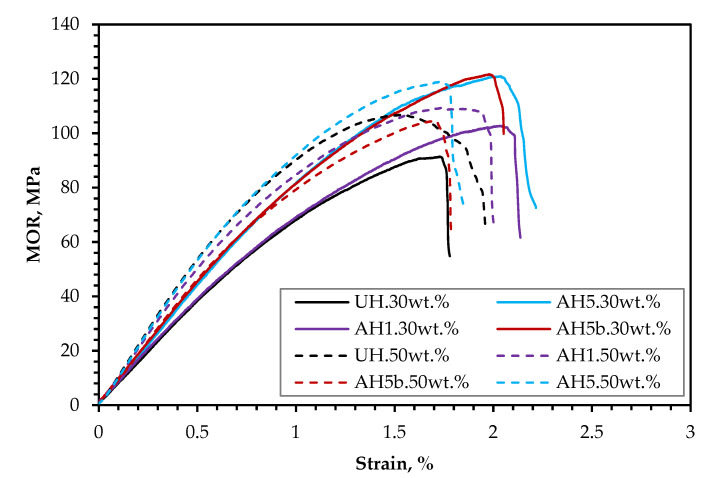
Flexural stress–strain curves of the biocomposites at 30 (–) and 50 wt.% (- -) fiber content.

Figure 10 also shows the flexural stress–strain curves similar to the tensile stress-strain curves in Figure 8. However, compared to the neat PLA, the elongation at the break of the biocomposites was also low, and there was similarly no noticeable yield or hardening stage.

In general, significant improvements were achieved in the mechanical properties of the biocomposites fabricated in this study compared to previously published results [17]. This superior performance could be credited to (i) improvement in the fabrication process, enabling improved fiber alignment from using smaller frames, since a 25% misalignment of the fibers was observed for the composites examined in the previous papers [11], (ii) laser cutting compared to machining with cutting blades that prevent straining or damaging the specimens before the mechanical test, (iii) using test specimen sizing, which reduces the impact of fiber non-uniformity and inhomogeneity within the composite network because the average length of the hemp fiber was determined to be 15 mm [17]. Compared to other relevant studies, Baghaei et al. [48] reported an increase in σ from (73 ± 6) to (77 ± 3) MPa for woven PLA yarns reinforced with 30 wt.% aligned (0⁰ to PLA yarn direction) after treating the hemp fibers for 1 h with 4 wt.% NaOH solution. The modulus also increased from (8.8 ± 1.4) to (10.3 ± 1.4) GPa. The mechanical test samples were also laser cut, but for direct comparison where non-woven PLA fibers are involved, the untreated hemp fiber reinforced PLA had a σ of (53 ± 1.2 MPa) and modulus of (5.6 ± 1.1 GPa). Furthermore, Islam et al. [46] reported a σ of 82.9 MPa, modulus of 10.9 GPa, MOR of 142.5 MPa and MOE of 6.5 GPa for composites of 30 wt.% long aligned hemp fibers treated with 5 wt.% NaOH solution and 2 wt.% Na_2_SO_3_ for 1 h.

#### 3.3.3. The Impact of FR Coating on Mechanical Performance

**Figure 11:**
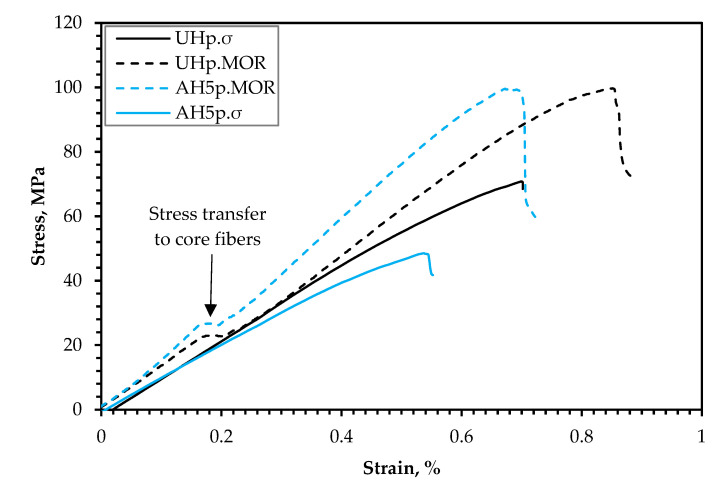
Stress (σ(–)];[MOR (- -))–strain curves of UHp and AHp.

The current study demonstrates that a 5 wt.% alkali treatment of the hemp fiber for 1 h appears to be the most efficient alkali treatment for overall improvement in biocomposite performance for the assessed frost-retted hemp fiber. Consequently, to investigate the mechanical performance following FR treatment, the biocomposites reinforced with 50 wt.% untreated hemp fibers coated with F1 (UHp) and hemp fibers treated with 5 wt.% alkali for 1 h and coated with F1 (AH5p) were studied. The tensile and flexural properties are also presented in Figure 7 and Figure 9. Figure 7a and Figure 9a show that the F1 treatment had a greater impact on biocomposite σ than MOR, which is not surprising because the whole cross-section of the sample is subjected to the highest stresses during the tension test; thus, the load applied to the specimen stays constant for all fibers in the composites. As a result, it is more probable to discover a weak point from which the failure occurs. Indeed, Figure 11 supports this reason because the tensile stress–strain curves (UHp.σ and AH5p.σ) seem similar to the curves presented in Figure 8 of the non-F1 composite batches, whereas the flexural stress–strain curves (UHp.MOR and AH5p.MOR) highlight an area suggesting stress transfer from the top fibers to the core fibers during flexural load. When the biocomposite is under flexural test, the stress occurs in the small area of the bending region and the load received by the biocomposite is transmitted by shear at the cross-sectional point of the test through the fibers. As the top fibers fail, the core non-F1-coated fibers receive the load and provide better support due to their superior strength properties. Conversely, the MOR for AHp was slightly better than UHp because the core hemp/PLA layer has better interfacial interaction, which ensures better shear transfer.

Comparing the σ result based on the introduction of alkali treatment, UHp and AH5p show a reduction of 14% and 42%, respectively, compared to the non-FR variants (UH and AH5). Despite the higher flexural performance for AH5p compared to UHp, the decrease in MOR relative to AH5 was considerably higher (8%) than for UHp compared to UH (6%). The results from Section 3.3.1 and Section 3.3.2 demonstrate that reinforcing PLA with hemp fiber generally improves mechanical performance compared to neat PLA. According to Islam et al. [46], the thermal characteristics of the hemp/PLA composite and PLA showed that crystallinity increased with the incorporation of hemp fiber compared to the neat PLA. This increase in crystallinity was ascribed to the hemp fiber’s nucleating ability, which improves after alkali treatment due to the removal of lignin. Therefore, the considerable reduction in σ for AH5p compared to UHp might be attributed to a more severe loss in crystallinity caused by fire retardant degradation of the cellulose in AH5p that is higher than in UHp.

**Figure 12:**
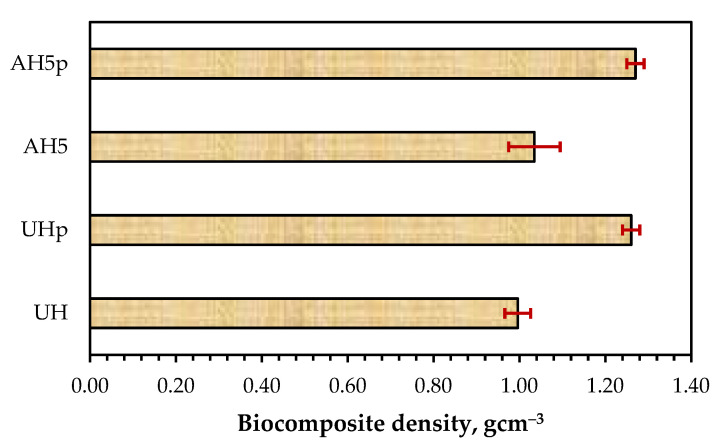
Density of biocomposites reinforced with 50 wt.% hemp fibers (untreated (UH), untreated with F1 (UHp), 1-h treatment with 5% alkali (AH5) and the variant with F1 (AH5p).

Figure 7b and Figure 9b also show that the *E* and MOE of UHp and AH5p were significantly higher than the non-FR batches. This increase in rigidity occurred due to the decrease in flexibility of the hemp fiber, as observed from the significant reduction in the biocomposite elongation at break (Table 3), the F1 coating, and the higher density of UHp and AHp compared to UH and AH5 (Figure 12). The biocomposite properties with regards to density, *E* and MOE were the same for UHp and AH5p.

### 3.4. Fire Performance

The fire behavior of the biocomposites was defined by (i) mass loss (m_loss_), (ii) time to ignition (t_ig_), (iii) surface temperature at ignition (Ig_temp_), and (iv) temperature response through depth (t_depth_). The potential of the biocomposite to function as a fire barrier was investigated using the basic protection time of a material (t_prot_) and the start time of charring of timber (t_ch_), which corresponded to through-depth temperatures of 270 and 300 °C (EN 1995-1-2:2004), respectively, as used in previous research [18,30]. 

**Figure 13:**
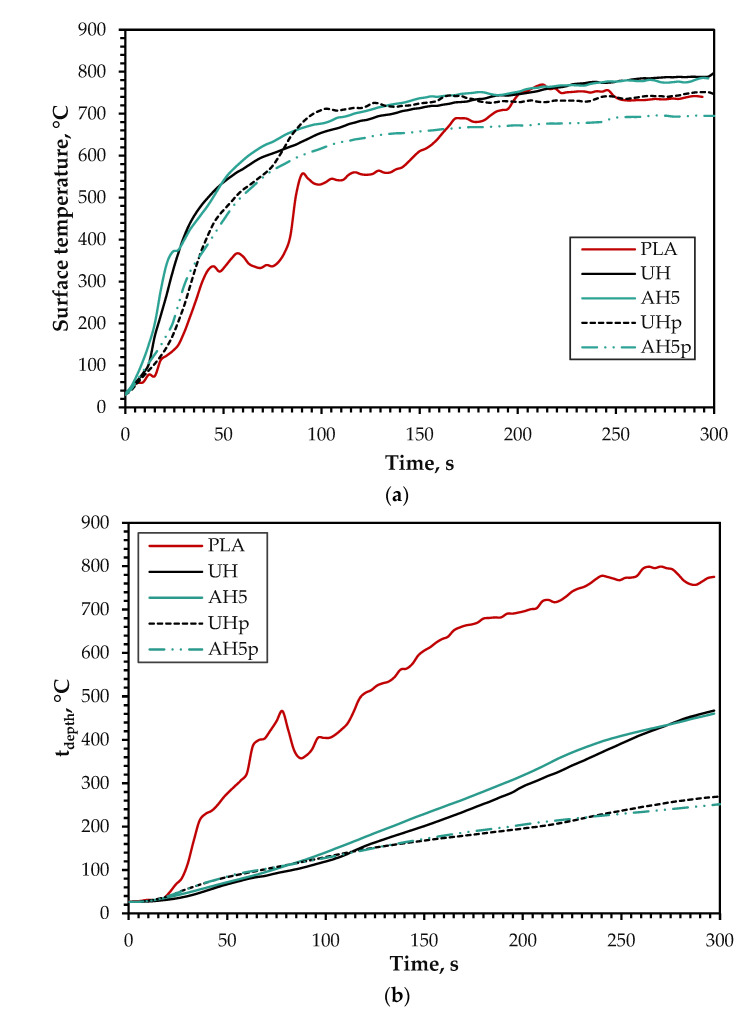
(**a**) Surface temperature(t_surf_) and (**b**) temperature transfer through depth(t_depth_) of neat PLA, UH, AH5, UHp and AH5p.

Figure 13a shows a delayed plateau at about 100 °C that is associated with the evaporation of moisture. This plateau was delayed more for the biocomposites with untreated hemp fibers (UH and UHp), which is indicative of the presence of higher moisture content due to their more amorphous nature compared to the alkali-treated variants. The temperature rise was the fastest for UH and AH specimens because of the fast t_ig_ (13 and 14 s, respectively), as shown in Table 4. However, when the neat PLA was subjected to heat from the cone heater, there was a slight delay in the ignition (27 s) associated with the deformation of PLA. Flaming was then initiated through the combustion of both the PLA and timber block, causing a high ig_temp_ (155 °C) compared to UH and AH5. The t_ig_ was similar for UH and AH5, but AH5 produced a higher ig_temp_ related to the higher cellulosic content.

Figure 13b shows that t_depth_ was the highest for the neat PLA, which is due to the direct combustion of the timber block after the thermal deformation of the PLA board, causing the low t_prop_ and t_ch_ shown in Table 4. Even though UH and AH5 ignited faster than the neat PLA, the t_depths_ were much lower, resulting in better t_prop_ and t_ch_. This outcome is due to the charring nature of the hemp fiber [6] that protects the timber from burning. Though no meaningful difference was observed between UH and AH5, the t_prop_ and t_ch_ were marginally higher for UH because of the higher lignin content that caused more charring during the fire exposure [6].

Regarding the F1 treatment, the coated biocomposites (UHp and AHp) had much-improved fire performance compared to UH and AH5. Figure 13a shows that though UHp and AH5p presented the same combustion pattern as UH and AH5, the surface temperature was lower because of delayed ignition or lack of it (see Table 5). Table 5 also shows that not all the tested samples from AH5p ignited after exposure to the cone heater, while the lowest t_ig_ and corresponding ig_temp_ were 96 s and 577 °C. Compared to AH5, this represents a remarkable improvement of 586 and 285%, respectively. In the case of UHp, all the tested samples ignited with an average t_ig_ and ig_temp_ of 35 s and 337 °C, respectively, which was a relatively significant improvement of 169 and 144% compared to UH. Despite the difference in ignition parameters, the t_depth_ was similar (Figure 13b) and much lower than those of UH, AH5 and the neat PLA, showing there was some protection for the timber during the test, as highlighted in Table 5.

**Figure 14:**
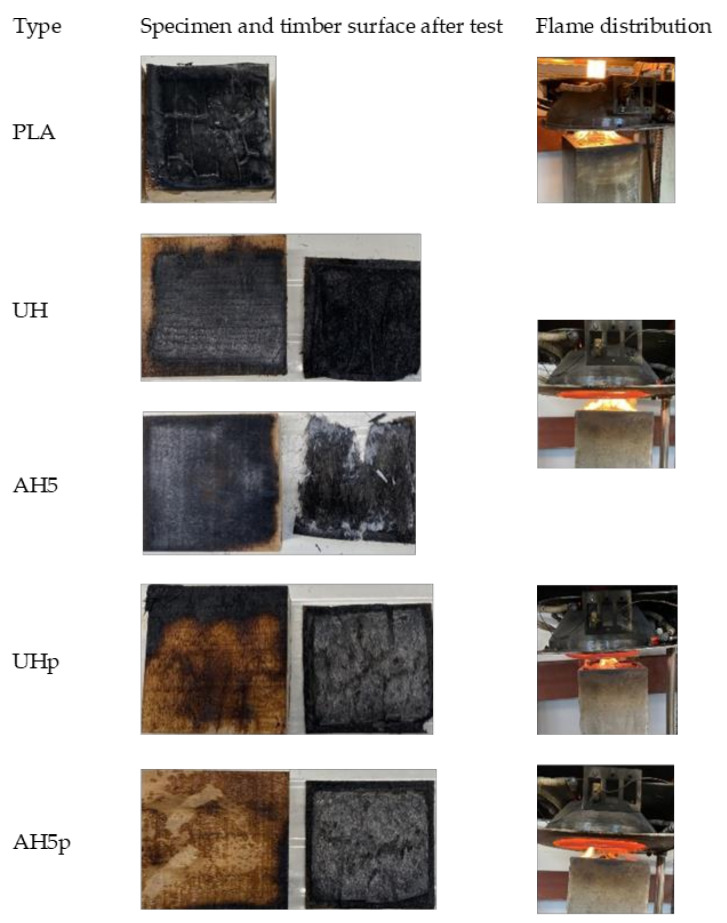
The timber block, specimen surface after the test, and flame distribution during the test.

**Figure 15:**
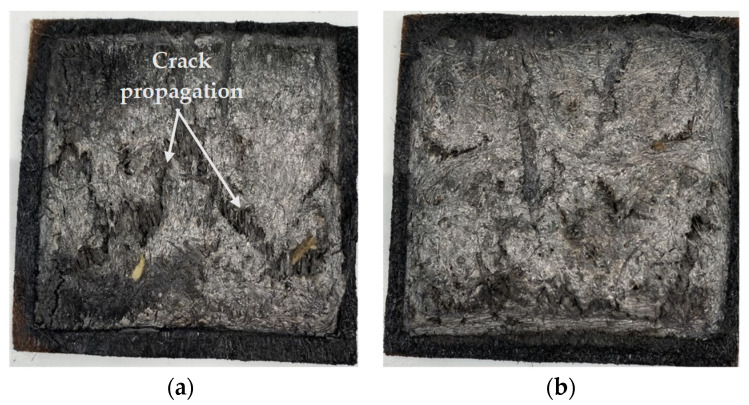
Outlook of the F1-coated biocomposites reinforced with (**a**) untreated and (**b**) alkali-treated hemp fibers after fire test.

Figure 14 shows the tested specimens, the surface of the timber block after the fire test, and the flame propagation during the experiment. The timber block was mostly protected and prevented from charring in the cases of AHp and UHp, which agrees with the fire performance data previously discussed. In the case of PLA, the surface of the timber was thoroughly charred, while the UH and AH5 showed similar outcomes in the flaming and on the surface of the underlying timber block. The fire intensity appears to be lowest in the case of the board variants with F1 treatment, but the neat PLA had the highest, while UH and AH did not show any difference in flame intensity. The flaming observed for the A5Hp sample in Figure 14 highlights a localized ignition, which may suggest that a non-uniform distribution of F1 on the hemp fiber surface could be the actual reason for the ignition. In the instance of UHp, the entire composite surface was split up during the test, as shown in Figure 15, revealing the core, untreated layer and possibly explaining the quick time to ignite, which could be due to debonding between the top and the middle mats. Furthermore, Table 5 shows that the F1 retained was significantly lower for UHp, although this appears to be far from the reason it ignited faster, given that UHp2 had higher F1 content than AH5p2, which did not ignite.

## 4. Conclusions

In this study, alkali concentrations of 1%, 3%, and 5% were used with varying treatment periods to extract non-cellulosic components from frost-retted hemp fiber. As measured by fiber mass loss, the quantity of non-cellulosic material removed increased with NaOH concentration and treatment duration. The elimination of these target components also caused alterations in the fiber structure, as demonstrated by FTIR spectroscopy. However, the fiber treatment with a 5 wt.% NaOH solution appeared to be the most successful, though there was no significant difference in the amount of non-cellulosic materials removed after 1, 2, and 4 h. At the composite level, it was discovered that treating hemp fiber for 1 h with a 5 wt.% NaOH solution is the most efficient technique for achieving the best overall mechanical performance. As predicted, fire retardant (Palonot F1) coating resulted in a considerable increase in biocomposite fire protection. While the biocomposite flexural properties were practically intact, the tensile strength, particularly for the alkali-treated batch, was reduced by 46%. This substantial decrease in tensile strength could be due to a more significant degradation of composite cellulosic characteristics than composites reinforced with FR-coated untreated hemp fibers.

This study demonstrates the ability to improve NFRC fire performance and the overall influence of combining fiber surface treatment and fire-retardant coating on the biocomposite’s mechanical and fire protection capabilities. Larger-scale fire experiments are required to validate results and offer further information.

## Figures and Tables

**Table 1:** The properties of hemp fiber used in this study.

Cellulose (%)	Hemicellulose (%)	Lignin (%)	Other Components (%)	Tensile Strength (MPa)	Modulus (GPa)
77.4 ± 0.3	8.3 ± 0.3	1.4 ± 0.0	12.9 ± 0.4	500 ± 239	16.6 ± 8.5

**Table 2:** Concentration of NaOH solution and duration of treatments examined.

Concentration	Durations, Hour(s)			
1 wt.%	-	1	2	4
3 wt.%	0.5	1	2	4
5 wt.%	0.5	1	2	4

**Table 3:** Comparing the strain at break of the FR-coated biocomposites with the non-FR-coated batches.

Composites	Strain at Break under Tensile Loading, %	Strain at Break under Flexural Loading, %
UH	1.2 ± 0.2	1.9 ± 0.3
UHp	0.6 ± 0.1	0.8 ± 0.1
AH	1.1 ± 0.2	2.0 ± 0.4
AHp	0.5 ± 0.1	0.7 ± 0.1

**Table 4:** Specimen’s mass loss, ignition time, temperature transfer through depth, basic protection time and start of char time.

Sample	M_loss_, %	t_ig_, s	Ig_temp_, °C	t_prop_, s	t_ch_, s
PLA	100 ± 0.0	27 ± 2.5	155 ± 18	45 ± 7.9	51 ± 10.8
UH	84 ± 1.8	13 ± 2.3	138 ± 26	194 ± 18.1	210 ± 21.0
AH5	84 ± 2.4	14 ± 1.7	150 ± 19	177 ± 27.5	192 ± 27.5

**Table 5:** Ignition parameters for UHp and AHp.

Sample	F1 Deposition, %	m_loss_, %	t_ig_, s	ig_temp_, °C	t_prop_, s	t_ch_, s
UHp1	21.46	50.56	40	281.60	-	-
UHp2	22.35	49.74	35	390.46	261	-
UHp3	20.58	47.11	30	339.20	-	-
Average	21.46	49.14	35	337.09	-	-
Std.	0.89	1.80	5.0	54.46	-	-
AH5p1	22.53	47.91	96	577.09	-	-
AH5p2	22.02	44.63	-	-	-	-
AH5P3	23.95	39.58	252	735.99	-	-
Average	22.77	44.04	-	-	-	-
Std.	0.83	4.19	-	-	-	-

## Data Availability

Data sharing not applicable.
